# RNA‐seq‐based selection of reference genes for RT‐qPCR analysis of pitaya

**DOI:** 10.1002/2211-5463.12678

**Published:** 2019-07-11

**Authors:** Quandong Nong, Yongchao Yang, Mingyong Zhang, Mei Zhang, Jiantong Chen, Shuguang Jian, Hongfang Lu, Kuaifei Xia

**Affiliations:** ^1^ Key Laboratory of South China Agricultural Plant Molecular Analysis and Genetic Improvement South China Botanical Garden Chinese Academy of Sciences Guangzhou China; ^2^ Wenshan Academy of Agricultural Sciences China; ^3^ Guangdong Provincial Key laboratory of Applied Botany South China Botanical Garden Chinese Academy of Sciences Guangzhou China

**Keywords:** gene expression, pitaya, reference gene, RNA‐seq, RT‐qPCR

## Abstract

Reverse‐transcription quantitative real‐time PCR (RT‐qPCR) is a primary tool for measuring gene expression levels, and selection of appropriate reference genes is crucial for accurate and reproducible results of gene expression under various experimental conditions. However, no systematic evaluation of reference genes in pitaya (*Hylocereus undatus* Britt.) has been performed. Here, we examined the expression of five candidate reference genes, namely elongation factor 1‐alpha (*HuEF1‐*α), 18S ribosomal RNA (*Hu18S rRNA*), ubiquitin (*HuUBQ*), actin (*HuACT*), and ubiquitin‐conjugating enzyme (*HuUQT*), under different conditions in pitaya. The expression stabilities of these five genes were evaluated using two computation programs: geNorm and NormFinder. The results were further validated by normalizing the expression of the phosphoglycerate kinase (*HuPGK*) and ethylene‐responsive transcription factor (*HuERF*) genes. Our results indicate that combined use of *HuUBQ* and *HuUQT* is the most stable reference under all of the experimental conditions examined. *HuEF1‐*α, *HuUBQ,* and *HuUQT* are the top three most stable reference genes under salt stress, drought stress, and heat stress, and across different cultivars. *HuEF1‐*α, *HuACT*, and *HuUQT* exhibited the most stable expression patterns across different tissues. Our results will allow researchers to select the most appropriate reference genes for gene expression studies of pitaya under different conditions.

**Abbreviations**
*18S rRNA*: 18S ribosomal RNA
*ACT*: actin
Ct: threshold cycle
*EF1‐*α: elongation factor 1‐alpha
E: primer amplification efficiency
Error: mean squared error of the single data points fit to the standard curve
FPKM: fragments per kilobase of transcript per million mapped reads
M: average expression stability
RT‐qPCR: reverse‐transcription quantitative real‐time PCR
SS: sum of squares of deviations
*UBQ*: ubiquitin
*UQT*: ubiquitin‐conjugating enzyme
V: pairwise variations

Reverse‐transcription quantitative real‐time PCR (RT‐qPCR) has become a critical technique for gene expression studies due to its rapidity, simplicity, reproducibility, specificity, and sensitivity 1, 2, 3. The reliability of RT‐qPCR data is affected by the reference gene and sample preparation 4. An ideal reference gene usually has stable expression level under different experimental conditions 5, 6, 7. However, an increasing number of studies have shown that none of the common reference genes meet this criterion for all of the test conditions. Their expression shows large variations in different tissues, developmental stages, and under different stresses 8, 9, 10, 11. Therefore, a suitable reference gene for various experimental conditions must be empirically selected before any reliable RT‐qPCR data can be obtained 12, 13.

In recent years, suitable reference genes for various experimental conditions have been studied in many species, such as rice 14, rubber tree 15, sunflower 16, black locust 17, grapevine 18, and annual ryegrass 19. Many reference genes have been identified in specific experimental conditions. For instance, elongation factor 1‐alpha (*EF1‐*α) was the best reference gene to normalize gene expression under heat stress, waterlogging, and across different tissues in *Amorphophallus* 20. Ubiquitin (*UBQ*) and actin (*ACT*) were the most stable reference genes in different developing seeds and cultivars in the tung tree 21, while *18S* ribosomal RNA (*18S rRNA*) was the optimum reference gene under the salinity and drought conditions in kenaf 22.

Pitaya (*Hylocereus undatus* Britt.) is a tropical and subtropical world fruit that is highly valued as a functional food 23, 24, and it usually refers to fruit of the genus *Hylocereus* of the Cactaceae family. This fruit is popular around the world, especially in Brazil, America, Russia, and Southeast Asia areas. In recent years, with the increase in population and the limited land resources, more and more studies have been focusing on the molecular mechanism of stress response in pitaya 25, 26, 27. Accurate assessment of gene expression changes using RT‐qPCR under these conditions is a rapid and effective approach to identify the stress‐responsive genes, which could also contribute to stress resistance breeding efforts. However, due to limitations in genomic sequencing data, only a few reference genes were cloned in pitaya, including *HuACT*,* HuUBQ*,* Hu18S rRNA*, and ubiquitin‐conjugating enzyme (*HuUQT*) (sequence for *Hu18S rRNA* not available) 28, 29. Furthermore, these genes have not been submitted to a systematic and accurate stable expression validation analysis under various experimental conditions in pitaya. In this study, the aim was to identify reliable reference genes that could serve as normalization factors for RT‐qPCR data analysis in pitaya. Five candidate reference genes (*HuACT*,* HuUBQ*,* Hu18S rRNA*,* HuUQT*, and *HuEF1‐*α) were selected, and we evaluated by RT‐qPCR their expression stability under salt stress, drought stress, and heat stress, in different cultivars and across different tissues. In addition, the expression levels of the phosphoglycerate kinase (*HuPGK*) and ethylene‐responsive transcription factor (*HuERF*) genes, which are upregulated in response to salt stress in pitaya 30, were measured to determine the reliability of our results using two programs (geNorm and NormFinder).

## Materials and methods

### Plant materials and treatments

Pitaya seeds were germinated and grown in a greenhouse under controlled conditions (14‐h photoperiod, 28 °C ± 1 °C, and 60% ± 5% relative humidity). Three‐month‐old seedlings were treated with 200 mm NaCl, high temperature (40 °C), and drought stress. For salt and drought treatments, the roots were collected before or after stress for 0, 3, 6, 24 h, and 3 days. For high‐temperature treatment, the stems were collected after 0, 3, 6, 24 h, and 3 days growing in a 40 °C growth chamber. Three‐month‐old stems were collected from cultivars of the South China Botanical Garden resource of pitaya germplasm (longitude: 113.35°; latitude: 23.17°), namely: ‘Zishan 1’, ‘Zishan 2’, ‘Meigui 1’, ‘Jingdu 1’, ‘Red pitaya 1’, and ‘Red pitaya 2’. Different tissues, including the root (3 months old), the stem (3 months old), petals (3 days after flowering), and the calyx (3 days after flowering), were collected separately at 10:00 a.m. For all of the treatments, there were three biological replicates for each sample. All of the samples were immediately frozen in liquid nitrogen after collection and stored at −80 °C until RNA extraction.

### RNA extraction and cDNA synthesis

Total RNA was extracted using Eastep® Super Total RNA Isolation Kit (Promega, Shanghai, China). Genomic DNA was removed by treatment with RNase‐free DNase I (Promega). The RNA concentration was determined with a NanoDrop 2000c Spectrophotometer (Thermo Fisher Scientific Inc., Waltham, MA, USA). Total RNA (1 μg) was used for reverse transcription with GoScript™ Reverse Transcription Mix‐Random Primers Kit (Promega) in a 20 μL reaction volume according to the manufacturer's manual. The synthesized cDNAs were verified by RT‐qPCR and diluted fivefold for RT‐qPCR analyses.

### Gene cloning

We used RNA‐seq *de novo* to assemble and characterize the transcriptomic profiles of red pitaya roots in response to salt stress. The cDNA from samples of 0, 3, 7, and 30 h under salt stress was sequenced with Illumina HiSeq platform 30. The clean high‐quality data were obtained by removing reads containing adapters or ploy‐Ns and low‐quality reads from the raw data. *De novo* assembly of the RNA‐seq reads was conducted for each sample by using the Trinity program 31 with default parameters. This program assembled the high‐quality RNA‐seq reads into sequences, clustered the sequences, and reported full‐length transcripts. Then, *de novo* assembly of the transcriptomes was performed using a contig assembly program (CAP3; https://www.msi.umn.edu/sw/cap3-contig) with parameters ‐p 99 ‐f 2 ‐o 100. Statistical analysis was performed using the NGS QC Toolkit v.2.3.3 with default parameters 32. *18S* *rRNA* sequence of *Arabidopsis thaliana* (NR_141642.1) and the *EF1‐*α sequence of *Oryza sativa* (KT862214.1) were used to blast the red pitaya root contig assembly to obtain the corresponding homologous gene sequences. The primers for cloning the fragments of *18S rRNA* and *EF1‐*α were designed using primer premier 5 (PREMIER Biosoft, Palo Alto, CA, USA) based on the homologous contig sequences. The amplified PCR products were sequenced by TsingKe Biological Technology Company (Beijing, China), and the genes were separately named *Hu18S rRNA* (GenBank: MK160494) and *HuEF1‐*α (GenBank: MK160495). All of the sequences of these two candidate reference genes are shown in Data S1.

### Primer design

Gene‐specific primers of the candidate reference genes were designed using primer premier 5 (Premier Biosoft) based on the results of sequencing and the published sequences 28, 29. The sizes of amplicons from the five candidate reference genes amplified by gene‐specific primers were between 100 and 250 bp in length (Table 1). Primer specificity was checked by melting curve and agarose gel electrophoresis analyses. The primer amplification efficiency (*E*) was obtained from a standard curve generated by five serial dilutions of cDNA and was calculated as follows: *E = (*10^−1/slope^ − 1) × 100% 33. Primers were only used in subsequent experiments if the condition 90% ≤ *E *≤* *110% was met 34, 35, and the mean squared error of the single data points fit to the standard curve (Error) value was below 0.2, as recommended by the LightCycler® 480 Software user manual.

**Table 1:** Primer sequences and amplicon characteristics of five candidate reference genes. The *E* and Error values were calculated by the LightCycler® 480 Software.

Name	Primer sequence (forward/reverse primer)	Amplicon length (bp)	*E* (%)	Error
*HuEF1‐*α	5′‐CTGGACAAATCGGAAACGGC‐3′ 5′‐ATGTCCCTCACAGCAAAACGA‐3′	237	95.2	0.0055
*HuACT*	5′‐CTTCCATACCAATGAATGAGGGC‐3′ 5′‐TGAGCGAGAAATTGTCCGTGAC‐3′	200	96.7	0.0045
*HuUBQ*	5′‐ACCACGAAGGCGAAGCACA‐3′ 5′‐AAGATTCAGGACAAGGAGGGGAT‐3′	141	98.1	0.0110
*Hu18S rRNA*	5′‐TTCCGATAACGAACGAGACC‐3′ 5′‐CTATCCCCAGCACGACGAA‐3′	234	94.0	0.0120
*HuUQT*	5′‐CAATGCTGGGCTCCACTGC‐3′ 5′‐AATAATGGGCCCTGCAGATAGC‐3′	184	98.5	0.0054

### RT‐qPCR analysis

Reverse‐transcription quantitative real‐time PCR were executed in 384‐well plates using Roche Light Cycler 480 Real‐time PCR System (Roche, Basel, Switzerland). The protocol was as described by Xia *et al*. 36 with minor modifications. The total volume comprised of 1 μL of diluted cDNA template, 2 μm of each primer, 5 μL of Hieff™ qPCR SYBR® Green Master Mix (Shanghai YeaSen Biotech, Shanghai, China), add ddH_2_O to 10 μL in total. The thermal cycling protocol was as follows: an initial denaturation at 95 °C for 5 min, followed by 40 cycles of denaturation at 95 °C for 10 s, annealing at 57 °C for 20 s, and an extension at 72 °C for 20 s. An additional temperature‐ramping step from 95 to 60 °C was used to produce a melting curve. Three technical replicates were used for each sample.

### Data analysis

The expression stability of five candidate reference genes under different experimental conditions was calculated using geNorm (version 3.5) and NormFinder (version 0.953) according to the user manuals. The expression stability of each candidate reference gene was compared and ranked by each program according to the threshold cycle (Ct) values.

### Normalization of *HuPGK* and *HuERF*

To validate the stability of candidate reference genes, the gene expression levels of *HuPGK* (GenBank: MK160496) and *HuERF* (GenBank: MK160487) were quantified in the same cDNA templates (0, 3, and 7 h) by RNA‐seq and RT‐qPCR using all of the candidate reference genes (*HuEF1‐*α, *HuACT*,* HuUBQ*,* Hu18S rRNA*, and *HuUQT*) separately and as a combination of multiple reference genes (*HuUBQ* +* HuUQT* and *HuUBQ* +* HuUQT* +* HuEF1‐*α) (the gene sequences and specific primers are shown in Data S2). The fragments per kilobase of transcript per million mapped reads (FPKM) of different time points in RNA‐seq were used as a control (Table S3). The gene expression patterns in different time points (3 and 7 h) were calculated by log_2_ (Fold change).

## Results

### Verification of the primer specificity and amplification efficiency

**Figure 1:**
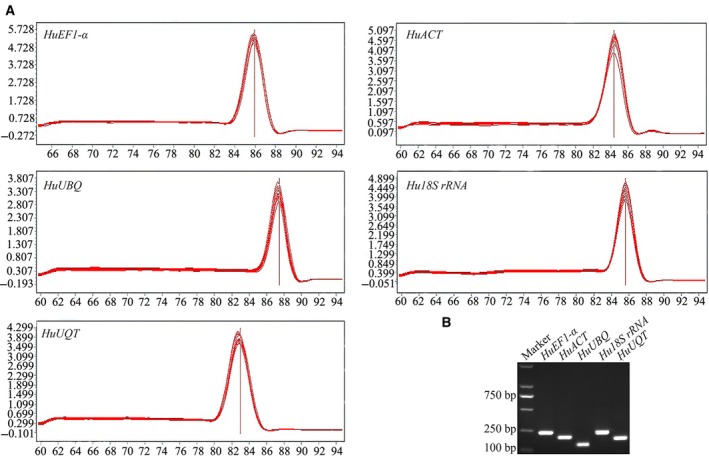
Specificity and amplification length of primers. The PCR amplification products for the five reference genes were analyzed by melting curves and agarose gel electrophoresis. (A) Melting curves for the PCR products. The single peak represents a specific PCR product. (B) Agarose gel electrophoresis. A single band with the anticipated product size indicates that the PCR product is specific.

To identify stable reference genes for gene expression studies in pitaya, five candidate reference genes were investigated by RT‐qPCR. The primer specificities of five candidate reference genes were detected based on melting curve and agarose gel electrophoresis analyses. The melting curve analysis shows that all of the primers amplified a single major peak (Fig. 1A) and a single PCR product of the expected size was observed on the gel (Fig. 1B). The *E* was calculated using a standard curve generated by five serial dilutions of cDNA by RT‐qPCR. The amplification efficiencies of all of the primers ranged from 94.0% to 98.5% and the Error values ranged from 0.0045 to 0.0120 (Table 1), which are both acceptable values.

### Expression profile of the candidate reference genes

**Figure 2:**
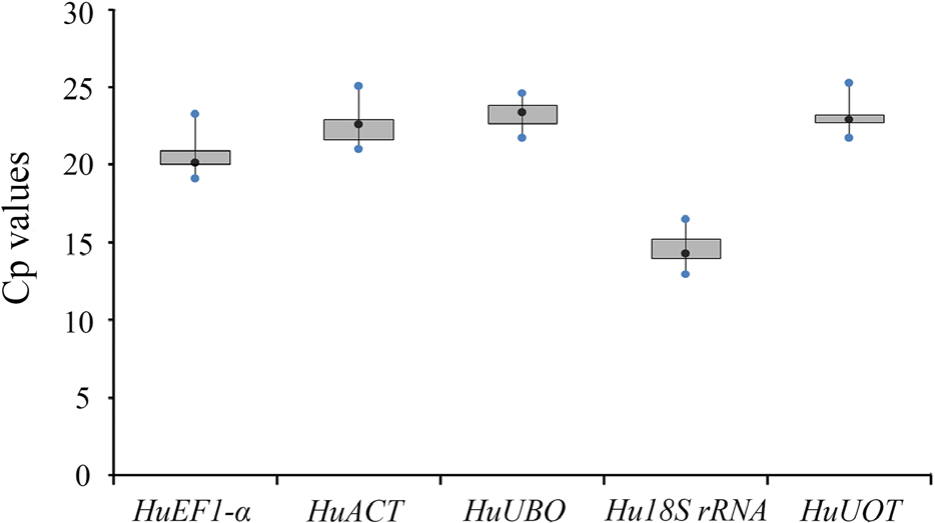
Crossing point (Cp) of five candidate genes across 78 cDNA samples in RT‐qPCR. The lower and upper ends of each box represent the 1/4 and 3/4 quartiles. Whiskers represent the maximum and minimum Cp values. The median Cp values are depicted by the dots in the boxes.

Using the LightCycler® 480 Software system, the Ct of a sample was identified as the point where the sample's fluorescence curve turns sharply upward. A lower Ct value indicates a higher expression level. In the present study, the Ct values for five candidate reference genes were determined for 78 samples, namely 18 samples from salt treatment, 15 samples from drought treatment, 15 samples from high‐temperature treatment, 18 samples of different cultivars, and 12 samples of different tissues. Five candidate reference genes had large variation in expression across all of the studied cDNA samples, and *HuUQT* was the gene with the smallest variation (Fig. 2). The Ct value ranged from 12.92 (*Hu18S rRNA*) to 25.26 (*HuUQT*), with the majority of these values being distributed from 21 to 24. *Hu18S rRNA* was the most abundant transcript (with lowest mean Ct value of 14.29) from the reference genes tested, while *HuUBQ* was the lowest expressed gene (with the highest mean Ct value of 23.39).

### Expressing stability of the reference genes

Two different software, NormFinder and geNorm, were used to analyze the gene expression stability of the candidate reference genes based on Ct values from different treatments and tissue sources. NormFinder algorithm calculated the inter‐ and intragroup variations of candidate reference genes and gave a stability value for each gene. The lowest stability value identifies the most stable expression. We found that *HuUBQ* and *HuUQT* presented the most stable expression in all of the tested experimental conditions. In contrast, *HuACT* (under salt stress, drought stress, and among different cultivars) and *Hu18S rRNA* (under heat stress and among different tissues) were the most unstable reference genes (Table 2).

**Table 2:** Stability of candidate reference genes calculated by NormFinder under different conditions. Lower stability value indicated the higher stable expression.

Rank	Salt stress		Drought stress		Heat stress		Different cultivars		Different tissues	
	Genes	Stability	Genes	Stability	Genes	Stability	Genes	Stability	Genes	Stability
1	*HuUQT*	0.25	*HuUQT*	0.12	*HuUQT*	0.15	*HuUQT*	0.12	*HuUBQ*	0.10
2	*HuUBQ*	0.41	*HuUBQ*	0.26	*HuUBQ*	0.23	*HuUBQ*	0.19	*HuUQT*	0.20
3	*HuEF1‐*α	0.58	*Hu18S rRNA*	0.34	*HuACT*	0.32	*Hu18S rRNA*	0.25	*HuACT*	0.53
4	*Hu18S rRNA*	0.62	*HuEF1‐*α	0.43	*HuEF1‐*α	0.41	*HuEF1‐*α	0.35	*HuEF1‐*α	0.58
5	*HuACT*	0.75	*HuACT*	0.86	*Hu18S rRNA*	0.58	*HuACT*	0.38	*Hu18S rRNA*	0.94

**Figure 3:**
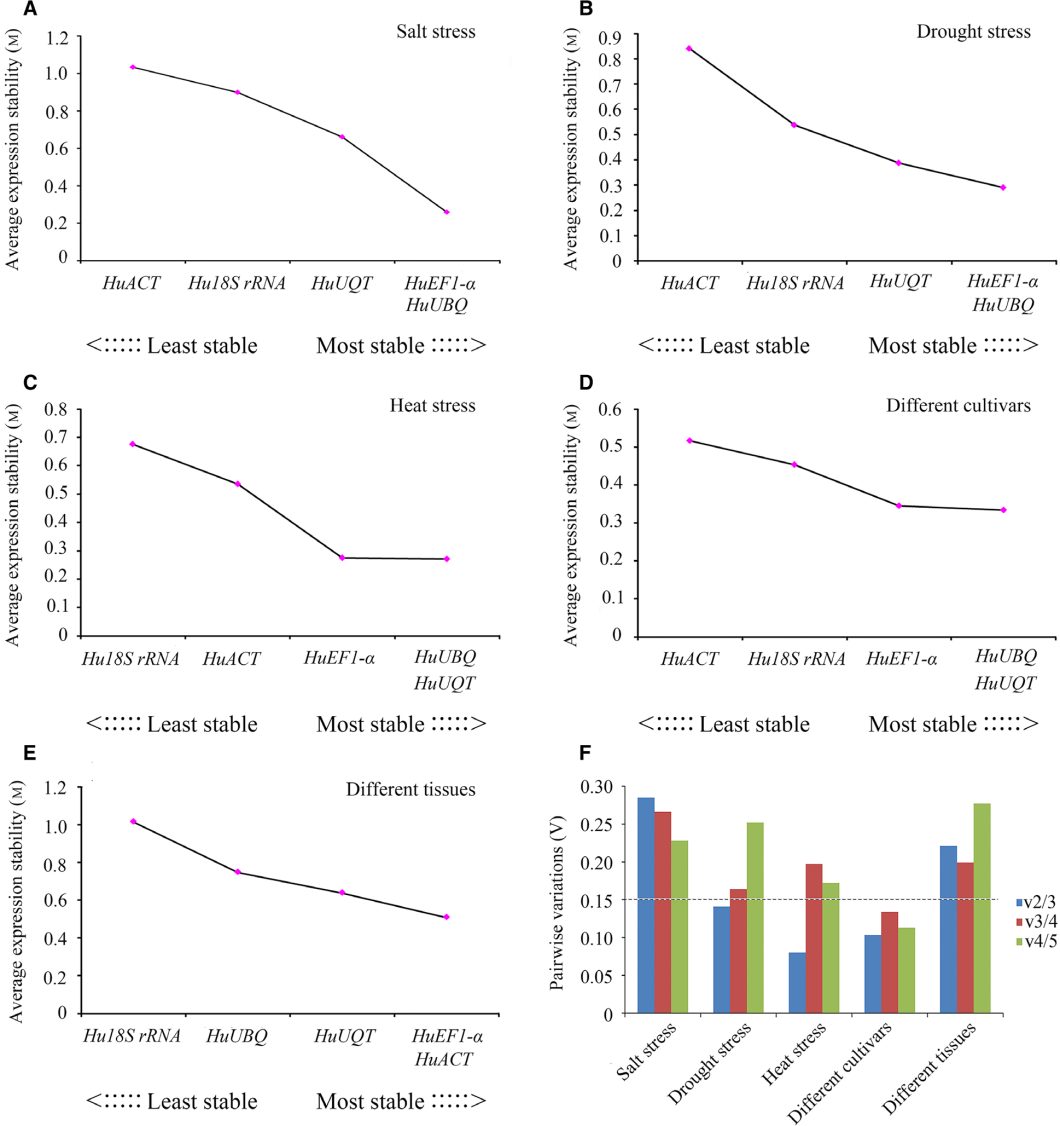
Average gene expression stability (M) and V analyses of the reference genes for different treatments and tissues, as determined using geNorm. Low M values indicate high expression stability. (A) Expression stability of five candidate reference genes in salt stress treatment. (B) Expression stability of five candidate reference genes under drought stress treatment. (C) Expression stability of five candidate reference genes under heat stress treatment. (D) Expression stability of five candidate reference genes under different cultivars treatment. (E) Expression stability of five candidate reference genes from different tissues. (F) The optimal number of reference genes required for effective normalization. The pairwise variation (*V*_*n*_/*V*_*n*+1_) was analyzed by the geNorm program to determine the optimal number of reference genes required for RT‐qPCR data normalization. 0.15 was used as a cutoff value, below which the inclusion of an additional reference gene is not required.

geNorm is another algorithm used to identify optimally stable reference genes through the average expression stability (*M*) and pairwise variations (V) values. Based on the geNorm analysis, all of the *M* values were under the threshold value of 1.5, that is, all of the candidate genes can be used as reference genes. However, *HuEF1‐*α and *HuUBQ* were the most stable reference genes under the salt and drought stresses, while *HuACT* was the most unstable gene (Fig. 3A,B). *HuUBQ* and *HuUQT* were the most stable reference genes under heat stress and among different cultivars, and *Hu18S rRNA* (under heat stress) and *HuACT* (among different cultivars) were the least stable genes (Fig. 3C,D). In different tissues, the genes displaying the most stable expression were *HuEF1‐*α and *HuACT*. On the contrary, *Hu18S rRNA* was the least stable reference gene (Fig. 3E).

According to the geNorm user manual, the pairwise analysis (*V*_*n*_/*V*_*n*+1_) is used to determine the optimal number of reference genes. Generally, 0.15 is used as a cutoff, which means that when *V*_*n*_/*V*_*n*+1_ <_ _0.15, it is not necessary to include more than reference genes. Under drought stress, heat stress, and among different cultivars, the *V*_2/3_ values were below 0.15 (Fig. 3F), therefore suggesting that the optimal number of reference genes is two genes. As the cutoff value of 0.15 is not absolute but rather a convention, an observed trend of changing *V* values is informative 6, 10. Also based on this criterion, we suggest that using two top stable reference genes as normalizing factors generates accurate data.

### Evaluation of normalization factors using *HuPGK* and *HuERF* RNA‐seq data

**Figure 4:**
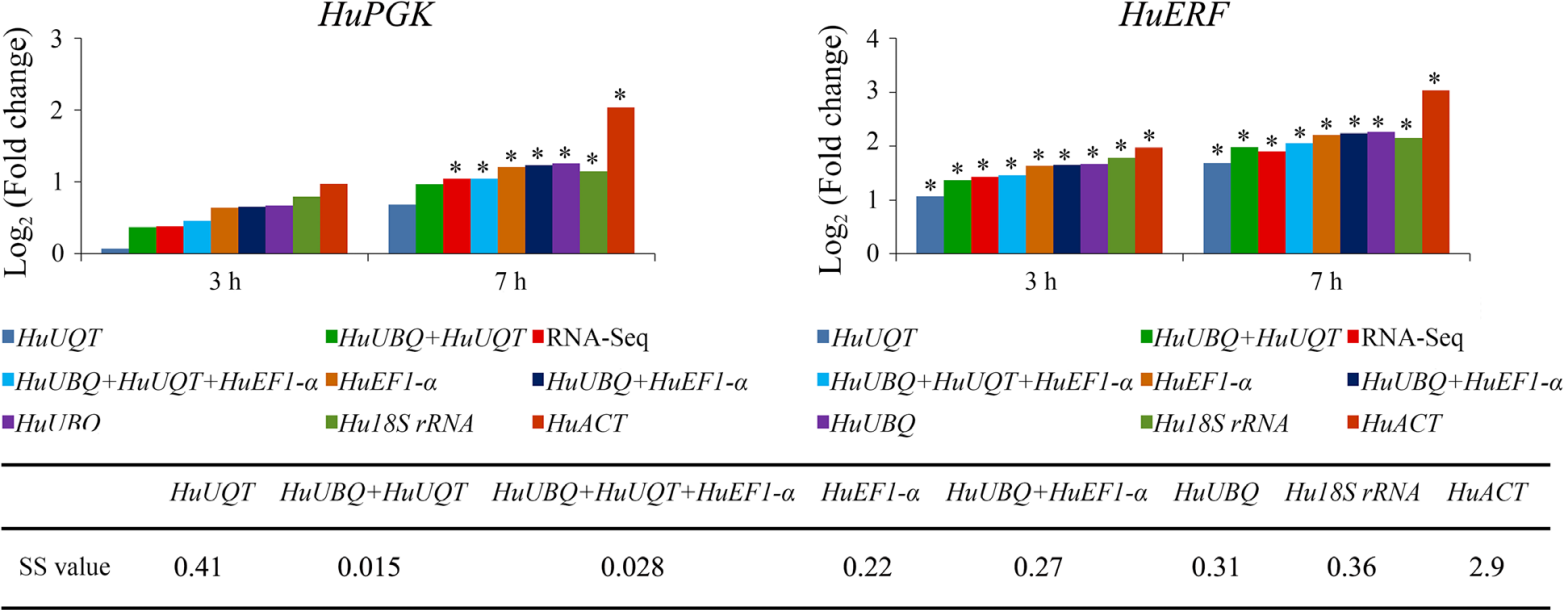
Validation of the reference genes by the relative expression of *HuPGK* and *HuERF* under salt stress. We selected all the reference genes and multiple top stable reference genes as normalization factors. The RNA levels of *HuPGK* and *HuERF* obtained from the RNA‐seq analysis are considered the most accurate measurement. ‘*’ represents the gene was upexpressed (log_2_ (Fold change) > 1). SS were calculated based on the average expression level normalized by eight normalization factors and the FPKM from RNA‐seq. The SS value is negatively correlated with the similitude between the qRT‐PCR and the RNA‐seq data, that is, the qRT‐PCR data with the lowest SS value are the most reliable.

*HuPGK* and *HuERF* were two upregulated genes in our RNA‐seq results. Their expression patterns in different time points under salt stress were analyzed using seven different normalization factors. The result showed that the estimation of the expression values of *HuPGK* and *HuERF* using RT‐qPCR data and different normalization factors was similar to estimation from the RNA‐seq data (Fig. 4). The lower the sum of squares of deviations (SS) value, the higher the similarity to the RNA‐seq data, and the results showed that SS values of normalization by a combination of the top stable reference genes were lower than the SS values of a single reference gene. In our study, the gene expression normalized by a combination of the most stable reference genes obtained from NormFinder, *HuUBQ* and *HuUQT,* was the most credible (with the lowest SS value of 0.015). For a single reference gene, *HuEF1‐*α and *HuUBQ* were the best reference genes, yielding SS values of 0.22 and 0.31, respectively. In contrast, *HuACT* was the worst reference gene in salt stress treatment. The stability ranking (*HuEF1‐*α > H*uUBQ* > *HuACT*) was similar to that of the geNorm, which indicates that the stability ranking by geNorm is more reliable than NormFinder.

## Discussion

Pitaya is a tropical fruit that is popular around the world due to its nutrition value and healthy properties 23, 24. There is a recent growing trend of studies in the literature focused on the molecular mechanism of stress response in pitaya, because the pitaya tree has a strong tolerance to drought, salt, and high temperature 37, 38, 39. The RT‐qPCR method has been widely used to analyze gene expression due to its rapidity, simplicity, reproducibility, specificity, and sensitivity. In these studies, it is vital to choose appropriate reference genes for obtaining accurate and reliable results. Four commonly used reference genes (*HuACT*,* HuUBQ*,* Hu18S rRNA*, and *HuUQT*) in pitaya studies have been cloned 28, 29. However, there has been no systematic and accurate validation of these genes under various experimental conditions. Here, we analyzed the expression stability of five commonly used reference genes under different experimental conditions and from different tissues.

Primer amplification efficiency indicates the amplicon doubling rate of a specific primer pair during a PCR, which affects the accuracy of RT‐qPCR data 4, 40. A good primer combination has an *E* value close to 100% 34, 35. In our study, the *E* values of the primers used to amplify the five candidate reference genes were between 94% and 98.5%. It ensured the accuracy of RT‐qPCR data in subsequent experiments.

*UBQ*,* UQT*,* ACT*,* EF1‐*α, and *18S rRNA* are traditional reference genes that are widely used in many plants. The expression stabilities of these genes were found to be different in different species as well as different experimental conditions in the same species 5, 9, 20, 22. In perennial ryegrass, *UBQ* and *UQT* were the best reference genes under waterlogging and heat stress, respectively 9, while *UBQ* was validated as one of the least stable in tea plan and black locust 5, 17. *UQT* was considered as an unstable reference gene in kenaf 22. *EF1‐*α was one of the stable expression genes in perennial ryegrass, *Amorphophallus*, and *Deschampsia antarctica* plants 7, 9, 20.

In our study, the expression stabilities of these genes in pitaya were analyzed by the geNorm and NormFinder computation programs. We found that the two algorithms produced the same ranking of the reference genes according to the differences in the stability (Table 2 and Fig. 3), which was consistent with previous studies 5, 19. The NormFinder analysis showed that *HuUBQ* and *HuUQT* were the two top stable reference genes in all of the experimental samples, while *HuACT* (under salt stress, drought stress, and different cultivars) and *Hu18S rRNA* (under heat stress and different tissues) were the most unstable reference genes. In the geNorm analysis, *HuEF1‐*α and *HuUBQ* were identified as the most stable reference genes under salt and drought stress, while *HuUBQ* and *HuUQT* were the most stable reference genes under heat stress and different cultivars. In different tissues, *HuEF1‐*α and *HuACT* were the most stable reference genes. Many studies revealed that using a combination of multiple top stable reference genes might improve the reliability of gene expression by RT‐qPCR 20, 41. In our study, the *V* values from geNorm calculated showed that a combination of the two top stable reference genes is the optimal solution.

In many studies, the expression of stable reference genes was evaluated by integrating multiple algorithms, including geNorm, NormFinder, BestKeeper, and comparative delta CT 8, 9, 19. Usually, a combination of genes was selected from the top stable genes in geNorm analysis 10, 18. However, in our study, we found that the gene expression patterns of *HuPGK* and *HuERF* were the most credible values when normalized by a combination of two top stable reference genes calculated by NormFinder (Fig. 4). Thus, we suggest that using two top stable reference genes calculated by NormFinder will produce the most accurate and reliable data. In contrast, in the comparison of single normalization factors, *HuEF1‐*α proved to be the best reference gene, followed by *HuUBQ*;* HuACT* was the worst reference gene. We further suggest that using the most stable gene ranked by geNorm will produce the most reliable data when using a single reference gene as the normalizing factor.

## Conclusion

In the present study, the expression of five candidate reference genes in pitaya under different conditions and from different tissues was compared and evaluated to identify stable reference genes for gene expression studies. Considering the geNorm and NormFinder analyses, the five candidate reference genes should be considered as reference genes in pitaya. Of these, the combination of *HuUBQ* and *HuUQT* was the optimal normalization factor under all of the tested experimental conditions. *HuEF1‐*α, *HuUBQ,* and *HuUQT* were the three top stable reference genes under salt stress, drought stress, heat stress, and different cultivars. With respect to different tissues, the three top stable reference genes were *HuEF1‐*α, *HuACT,* and *HuUQT*. Our results will be useful for future works involving gene expression analysis in pitaya using RT‐qPCR.

## Conflict of interest

The authors declare no conflict of interest.

## Author contributions

QN designed and performed the experiments, analyzed the data, and wrote the manuscript. YY helped in data analysis using the geNorm software. MZ helped to edit the manuscript. MZ and JC helped during sample collection. SJ and HL helped in the analysis of data. KX contributed to the experimental design and edition of the manuscript.

## Supporting information


**Data S1.** The sequences of *HuEF1‐*α and *Hu18S rRNA*.

**Data S2.** The sequences and specific primers of *HuPGK* and *HuERF*.

**Data S3.** The fragments per kilobase of transcript per million mapped reads (FPKM) of *HuPGK* and *HuERF* obtained from RNA‐seq.

## Data Availability

The RNA‐seq data are available at DDBJ database (BioSample Accession no.: SAMD00115774–SAMD00115785).
