# Supplementary material for: RNA‐seq‐based selection of reference genes for RT‐qPCR analysis of pitaya
Source: FEBS Open Bio. 2019 Jul 11;9(8):1403–12. doi: 10.1002/2211-5463.12678 (PMC6668369; doi:10.1002/2211-5463.12678)
Supplement: Supplementary file 3 — Data S3. The fragments per kilobase of transcript per million mapped reads (FPKM) of HuPGK and HuERF obtained from RNA‐seq. [file FEB4-9-1403-s003.docx]

| Gene | Transcript | FPKM | | |
| --- | --- | --- | --- | --- |
|  |  | 0 h | 3 h | 7 h |
| *HuPGK* | *Contig29387* | 9627 | 12560 | 19905 |
|  | *Contig29388* | 11183 | 14757 | 22945 |
| *HuERF* | *Contig26610* | 11428 | 30868 | 42718 |

**Table S3 The fragments per kilobase of transcript per million mapped reads (FPKM) of *HuPGK* and *HuERF* obtained from RNA-seq**
